# Evolution of the *NANOG * pseudogene family in the human and chimpanzee genomes

**DOI:** 10.1186/1471-2148-6-12

**Published:** 2006-02-09

**Authors:** Daniel J Fairbanks, Peter J Maughan

**Affiliations:** 1 Department of Plant and Animal Sciences, Brigham Young University, Provo, UT 84602, USA

## Abstract

**Background:**

The *NANOG *gene is expressed in mammalian embryonic stem cells where it maintains cellular pluripotency. An unusually large family of pseudogenes arose from it with one unprocessed and ten processed pseudogenes in the human genome. This article compares the *NANOG *gene and its pseudogenes in the human and chimpanzee genomes and derives an evolutionary history of this pseudogene family.

**Results:**

The *NANOG *gene and all pseudogenes except *NANOGP8 *are present at their expected orthologous chromosomal positions in the chimpanzee genome when compared to the human genome, indicating that their origins predate the human-chimpanzee divergence. Analysis of flanking DNA sequences demonstrates that *NANOGP8 *is absent from the chimpanzee genome.

**Conclusion:**

Based on the most parsimonious ordering of inferred source-gene mutations, the deduced evolutionary origins for the *NANOG *pseudogene family in the human and chimpanzee genomes, in order of most ancient to most recent, are *NANOGP6*, *NANOGP5, NANOGP3*, *NANOGP10*, *NANOGP2*, *NANOGP9*, *NANOGP7*, *NANOGP1*, and *NANOGP4*. All of these pseudogenes were fixed in the genome of the human-chimpanzee common ancestor. *NANOGP8 *is the most recent pseudogene and it originated exclusively in the human lineage after the human-chimpanzee divergence. *NANOGP1 *is apparently an unprocessed pseudogene. Comparison of its sequence to the functional *NANOG *gene's reading frame suggests that this apparent pseudogene remained functional after duplication and, therefore, was subject to selection-driven conservation of its reading frame, and that it may retain some functionality or that its loss of function may be evolutionarily recent.

## Background

Processed pseudogenes are derived from reverse transcription of RNA molecules followed by insertion of DNA copies into the genome. Therefore, for a processed pseudogene to be inherited from one organismal generation to the next, it must be derived from RNAs encoded by genes expressed in cells of the germline or the embryonic precursors of these cells. The homeobox gene *NANOG *is expressed in mammalian embryonic stem cells where its product, a homeobox transcription factor, maintains pluripotency of these cells [1-3]. Therefore, *NANOG *is an excellent candidate as a possible source of inherited processed pseudogenes. In fact, ten processed pseudogenes derived from *NANOG *are present in the human genome, an unusually large family of inherited processed pseudogenes derived from a single gene [4-6].

The human chromosomal region containing the *NANOG *gene has also undergone a tandem duplication resulting in two copies of the *NANOG *gene on chromosome 12. The two copies are approximately 97% identical and their transcripts are spliced differently [4,5]. Although there is EST-based evidence that both copies are transcribed, Booth and Holland [4] have argued that one of the two copies is an unprocessed pseudogene, which they named *NANOGP1*. They named the ten processed pseudogenes *NANOGP2 *through *NANOGP11*. Two are located on the X chromosome, two on chromosome 6, and one each on chromosomes 2, 7, 9, 10, 14, and 15. *NANOGP2 *and *NANOGP4 *through *NANOGP10 *are full-length or nearly full-length processed pseudogenes lacking introns. *NANOGP3 *and *NANOGP11 *are truncated fragments of processed pseudogenes [4,6].

Studies of unprocessed pseudogene evolution in primates are abundant, dating back to the early 1980s [7-9]. Although several studies are directed at pseudogene families [4,6,10], most focus on the evolution of a single processed pseudogene [11-14]. The relatively large number of processed *NANOG *pseudogenes and the recent release of the Build 1.1 assembly of the chimpanzee (*Pan troglodytes*) genome [15] provide an excellent opportunity to elucidate the evolutionary history of the *NANOG *gene and its large pseudogene family. This article compares the human *NANOG *gene and its pseudogenes with their chimpanzee orthologues and from this comparison derives an evolutionary history of this pseudogene family.

## Results and discussion

We identified the chimpanzee orthologues of the human *NANOG *gene and all of its pseudogenes except *NANOGP8 *using MEGABLAST and BLASTN searches of the Build 1.1 version of the chimpanzee genome assembly. Table 1 summarizes the chromosomal and genomic locations of the human *NANOG *gene and pseudogenes and their chimpanzee orthologues. MEGABLAST and BLASTN searches of the chimpanzee genome did not reveal any other *NANOG *sequences, suggesting that no new *NANOG *pseudogenes have arisen in the chimpanzee lineage since it diverged from the human lineage. However, we cannot rule out the possibility that additional *NANOG *pseudogenes may be present in the chimpanzee genome because unsequenced gaps remain in the Build 1.1 assembly. Our data indicate that the *NANOG *gene and all pseudogenes except *NANOGP8 *are in their expected orthologous positions in the chimpanzee genome, and that *NANOGP8 *is not present in the chimpanzee genome.

**Table 1:** Chromosomal locations and GenBank accessions of the *NANOG *gene and pseudogenes in the human and chimpanzee genomes.

Gene/Pseudogene	Human Chromosome and Genomic Location	Chimpanzee Chromosome and Genomic Location
*NANOG*	12 [GenBank:NT_009714] 12673–19336	12 [GenBank:DQ179631]
*NANOGP1*	12 [GenBank:NT_009714] 115849–122176	12 [GenBank:NW_114668.1] 785041–791563
*NANOGP2*	2 [GenBank:NG_004099]	2B [GenBank:NW_104777.1] 91227–92720, [GenBank:DQ301864]
*NANOGP3*	6 [GenBank:NG_004095]	6 [GenBank:NW_107947.1] 7895540–7895957, [GenBank:DQ301865]
*NANOGP4*	7 [GenBank:NG_004100]	7 [GenBank:NW_108883.1] 20417714–20419437, [GenBank:DQ301866]
*NANOGP5*	9 [GenBank:NG_004101]	9 [GenBank:NW_111809.1] 3226494–3228690
*NANOGP6*	10 [GenBank:NG_004102]	10 [GenBank:NW_113009.1] 12359797–12360738, [GenBank:DQ301867]
*NANOGP7*	14 [GenBank:NG_004098]	14 [GenBank:NW_115886.1] 751742–753321, [GenBank:DQ301868]
*NANOGP8*	15 [GenBank:NG_004093]	Absent
*NANOGP9*	X [GenBank:NG_004097]	X [GenBank:NW_121850] 2623421–2625487, [GenBank:DQ301869]
*NANOGP10*	X [GenBank:NG_004096]	X [GenBank:NW_121732.1] 2217356–2218968, [GenBank:DQ301870]
*NANOGP11*	6 [GenBank:NG_004103]	6 [GenBank:NW_107982.1] 2653050–2653450, [GenBank:DQ301871]

### Chimpanzee orthologues of *NANOG *and *NANOGP1*

*NANOG *[GenBank:NM_024865] is the functional gene in the human genome, whereas *NANOGP1 *[GenBank:AK097770] is apparently an unprocessed pseudogene derived from tandem duplication of the chromosomal region containing *NANOG*. However, cDNA and EST data show that *NANOGP1 *may be transcriptionally active, albeit at a lower level than *NANOG*, and that its transcripts are spliced differently than those derived from *NANOG*. Hart et al. [5] designated *NANOGP1 *as *NANOG2 *and referred to it as a functional gene, whereas Booth and Holland [4] argued that because of its relatively high degree of divergence from *NANOG*, and the comparative paucity and ambiguity of transcripts derived from it, *NANOGP1 *is an unprocessed duplication pseudogene.

MEGABLAST searches of the chimpanzee genome readily identified the orthologues of *NANOG *and *NANOGP1*. However, the organization of the chimpanzee orthologue of the human *NANOG *gene in the chimpanzee Build 1.1 genome assembly suggests that the gene is either rearranged in the chimpanzee genome, or that the assembly is incorrect within this gene. All four exons of the orthologue are present in the assembly but in two different GenBank accessions. The entire sequences of the 5' UTR, exon 1, and exon 2 are found in the region spanning nucleotides 683046 through 686855 of the chromosome 12 contig [GenBank:NW_114668], in a region on the short arm of chromosome 12 near the telomere at a location orthologous to that of the human *NANOG *gene at 12p13.31. Introns 1 and 2 of the chimpanzee orthologue are also within this region but large segments of them are unsequenced. The complete sequences of exon 3, intron 3, exon 4, and the 3' UTR of the chimpanzee orthologue are found in nucleotides 3808 though 5350 of another accession [GenBank:NW_115304], which is known to reside on chromosome 12 but has not been placed in the Build 1.1 assembly of this chromosome. Furthermore, exon 4 in this accession contains an apparent single nucleotide-pair insertion mutation, resulting in a frameshift and premature termination codon in the reading frame.

To determine if the apparent gene rearrangement and frameshift mutation are present in the chimpanzee *NANOG *gene, or whether these are assembly and sequencing errors, we compared the available sequences of *NANOG *and *NANOGP1 *in the chimpanzee assembly and selected PCR primer sequences in regions that differed sufficiently to ensure specific amplification of the *NANOG *gene. To verify that the amplicons were not derived from processed *NANOG *pseudogenes, all target sequences included at least a portion of a *NANOG*-specific intron.

Two primer combinations amplified fragments that include the region of apparent misassembly within intron 2. Both of these primer combinations amplified PCR fragments of the sizes expected if the gene is intact. We sequenced these fragments (and all other amplified fragments) of the gene and found that their sequences most closely matched those of the intact human *NANOG *gene and less closely the corresponding sequences in the human pseudogenes, including *NANOGP1*, confirming that our sequences are derived from the intact chimpanzee *NANOG *gene. Furthermore, our sequences show that the apparent frameshift mutation in exon 4 in the Build 1.1 assembly is a sequencing error. Our sequencing enabled us to assemble and annotate the genomic sequence of the intact chimpanzee *NANOG *gene [GenBank:DQ179631].

### Human *NANOGP8 *and its absence in the chimpanzee genome

**Figure 1:**
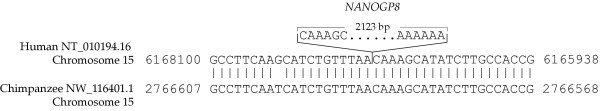
**Evidence that the *NANOGP8 *pseudogene is absent from the chimpanzee genome**. Sequences flanking the human *NANOGP8 *pseudogene on chromosome 15 are present in chromosome 15 of the chimpanzee genome but the pseudogene is absent. Comparison of the human and chimpanzee sequences shows that the *NANOGP8 *pseudogene inserted itself into human chromosome 15 without duplication of the surrounding sequences.

Human *NANOGP8 *[GenBank:NG_004093] is located on human chromosome 15 at 15q13.3. It is the most recent of the *NANOG *processed pseudogenes and is the only one that carries an *Alu *element found in the 3' UTR of the human *NANOG *gene. MEGABLAST and BLASTN searches of the chimpanzee genome failed to reveal the presence of a *NANOGP8 *orthologue; all significant hits were to the *NANOG *gene and other *NANOG *pseudogenes. To determine whether or not *NANOGP8 *is indeed absent from the chimpanzee genome, we used 762 nucleotides flanking the 5' end and 458 nucleotides flanking the 3' end of the human *NANOGP8 *pseudogene as queries in a BLASTN search of the chimpanzee genome. The search identified highly homologous and contiguous sequences on chimpanzee chromosome 15, spanning nucleotides 2765812 through 2767049 of the chromosome 15 contig [GenBank:NW_116401.1]. As shown in Figure 1, the *NANOGP8 *gene is indeed absent from its predicted site in the chimpanzee genome.

### Other *NANOG *pseudogenes in the chimpanzee genome

We identified the chimpanzee orthologues of the human *NANOG *processed pseudogenes *NANOGP2*, *NANOGP3*, *NANOGP4*, *NANOGP5*, *NANOGP6*, *NANOGP7*, *NANOGP9*, *NANOGP10*, and *NANOGP11 *in the Build 1.1 assembly. All of these pseudogenes are in their predicted chromosomal locations when compared to the human genome. The complete sequences of all of these pseudogenes except *NANOGP5 *and *NANOGP9 *are present in the chimpanzee genome assembly. A 100-nucleotide segment in the 3' UTR of *NANOGP5 *and a 1760 nucleotide segment containing the 5' UTR and the entire reading frame of *NANOGP9 *are unsequenced in the Build 1.1 assembly. However, the presence of 454 nucleotides of the 3' UTR, as well as orthologous flanking sequences, confirm the presence of *NANOGP9 *at its expected position.

We attempted to amplify the chimpanzee orthologue of *NANOGP9 *with primers designed to include small regions of flanking sequence on both ends to fully place it within the genome assembly. This pseudogene is embedded in repetitive sequences and, although our primers were designed to match what appeared to be small regions of nonrepetitive sequences in the flanking regions, they failed to amplify the target sequence. We designed primers to match unique sequences near the ends of the *NANOGP9 *reading frame (based on the human sequence) and successfully amplified and sequenced a region in *NANOGP9 *corresponding to positions 43–841 of the 918 nucleotide-pair reading frame in the functional *NANOG *gene [GenBank:DQ301869]. We verified that the sequence is indeed from *NANOGP9 *by its high similarity to the human orthologue. This sequence further confirms the presence of *NANOGP9 *in the chimpanzee genome and it allowed us to compare the sequences of the human and chimpanzee orthologues.

This sequence also resolved a question about the origins of *NANOGP9 *and *NANOGP10*. Both are located on the X chromosome and both contain a 15 nucleotide-pair deletion that does not appear in the alignment when these two pseudogenes are aligned with each other, suggesting that they share this deletion. These observations imply that *NANOGP9 *and *NANOGP10 *may be the products of a single insertion event followed by duplication of the chromosomal segment containing the pseudogene. However, these deletions reside in a region consisting of ten copies of an imperfect 15 nucleotide-pair tandem repeat within the reading frame. The chimpanzee *NANOGP9 *orthologue does not contain the deletion present in the human orthologue, whereas the chimpanzee and human orthologues of *NANOGP10 *have the same deletion. This observation indicates that the deletion in human *NANOGP9 *occurred after the H/C divergence and its origin is thus independent of the deletion in *NANOGP10*. Furthermore, we examined 5000 nucleotides on both sides of these pseudogenes and found no evidence of a duplication. We conclude that *NANOGP9 *and *NANOGP10 *originated independently.

### Evolution of the *NANOG *gene and pseudogene family

The entire functional *NANOG *gene (according to our sequencing data) and *NANOGP1 *are present in both the human and chimpanzee genome assemblies at orthologous chromosomal positions. In the 3' UTR of the *NANOG *gene, there is an *Alu *element, which is missing from *NANOGP1 *in both genomes. Therefore, the *NANOGP1 *unprocessed pseudogene arose through duplication of the chromosomal region containing *NANOG *before the human-chimpanzee (H/C) divergence and before insertion of the *Alu *element into the *NANOG *gene. Because the same *Alu *element is present in both the human and chimpanzee *NANOG *genes, its insertion must also have preceded the H/C divergence. The processed pseudogenes *NANOGP2*, *NANOGP3*, *NANOGP4*, *NANOGP5*, *NANOGP6*, *NANOGP7*, *NANOGP9*, and *NANOGP10 *lack this *Alu *element. They thus likely arose before its insertion and, therefore, also predate the H/C divergence. The presence of the *NANOGP11 *pseudogene fragment in both the human and chimpanzee genomes likewise shows that its origin preceded H/C divergence.

The human *NANOGP8 *pseudogene is highly similar to the *NANOG *gene, is absent from the chimpanzee genome, and contains the same *Alu *element as the *NANOG *gene, indicating that this processed pseudogene is the most recent of the *NANOG *pseudogenes and was inserted into human chromosome 15 after the H/C divergence.

Based on the assumption of a pseudogene mutation rate of 1.25 × 10^-9 ^mutations per site per year in humans [16,17], Booth and Holland [4] estimated the origin of the *NANOGP8 *pseudogene as the most recent at 5.2 million years ago, about the time of the H/C divergence. Our results demonstrate that *NANOGP8 *arose after the H/C divergence, and thus are consistent with this date. Booth and Holland [4] estimated the origins of the other pseudogenes as ranging from over 150 million years ago for *NANOGP6 *to 22 million years ago for *NANOGP1*, with the caveat that these dates may be inaccurate, and are likely overestimates, because nucleotide substitution rates for pseudogenes are not well calibrated within this range.

Booth and Holland [4] determined the relative ages of the human *NANOG *pseudogenes by counting the number of mutations in the reading-frame regions of the human *NANOG *pseudogenes when compared to the reading frame of the functional *NANOG *gene, scaling their analysis by counting adjacent deletions as a unit-site size of one to compensate for the reduced opportunity of substitution mutation in deleted regions. They concluded that *NANOGP6 *is the most ancient of the pseudogenes, followed in order of most ancient to most recent by *NANOGP5 *or *NANOGP3*, then *NANOGP10*, then *NANOGP9 *or *NANOGP2*, then *NANOGP7*, then *NANOGP4*, then *NANOGP1*, and *NANOGP8 *as the most recent. Booth and Holland's analysis did not distinguish the order of *NANOGP5 *and *NANOGP3 *relative to each other, nor of *NANOGP2 *and *NANOGP9 *relative to each other, because of similar degrees of divergence for each of these pairs of pseudogenes from *NANOG*.

We conducted a similar analysis of relative age, with the same scaling for multiple-nucleotide deletions as a single unit site when those deletions were shared by the human and chimpanzee sequences. We identified mutations that occurred after the H/C divergence as differences between the human and chimpanzee sequences and corrected them to reflect the ancestral sequence at the time of the H/C divergence before completing our analysis. This correction was especially important for *NANOGP10*, which has accumulated 20 mutations since the H/C divergence, compared to 1–10 mutations for the other pseudogenes. We excluded *NANOGP8 *from this correction because of its absence in the chimpanzee genome. Also, since *NANOGP3 *is a truncated pseudogene with only 254 nucleotides within the *NANOG *coding region, we compared only the portions of *NANOG *and the other pseudogenes that aligned with these 254 nucleotides when determining the relative age of *NANOGP3*. The pseudogene fragment *NANOGP11 *was not included in Booth and Holland's analysis nor ours because it lacks the entire reading frame and has no significant homology with several of the other processed pseudogenes.

Comparison of the sequences after these adjustments results in a relative order that is the same as that determined by Booth and Holland [4]. Also similar to Booth and Holland's conclusions, our analysis showed that *NANOGP3 *and *NANOGP5 *were almost identical in the degree of similarity to *NANOG *(88.6% and 88.2%, respectively), and that *NANOGP2 *and *NANOGP9 *were likewise nearly identical in the degree of divergence from *NANOG *(94.6% and 94.4%, respectively). Thus, like Booth and Holland [4], we could not conclusively determine the relative orders within each of these two pairs of pseudogenes using this type of analysis.

Such an analysis assumes that natural selection has conserved the functional gene's sequence so that the modern sequence of the reading frame represents the source sequence of each of the pseudogenes. Under most circumstances, such an assumption cannot readily be tested. However, the periodic insertion and fixation of ten *NANOG *pseudogenes with a complete or partial reading frame should have left a record, albeit an imperfect one, of the functional *NANOG *gene-sequence evolution. If we assume that the reading frame of the functional *NANOG *gene has changed during the time when the pseudogenes were inserted into the genome, the mutational differences in the pseudogenes should consist of three different types: 1) source-gene mutations, defined as those that occurred in the functional *NANOG *gene after the insertion of one pseudogene but before the insertion of another, resulting in a polymorphism between these pseudogenes, 2) post-insertion mutations, defined as those that occurred in a pseudogene after its insertion but before the H/C divergence, and 3) post-H/C divergence mutations, defined as mutations that occurred in the *NANOG *gene and its pseudogenes after the H/C divergence. We readily identified 88 post-H/C divergence mutations in the reading-frame regions of the *NANOG *gene and its pseudogenes, and in all but four cases we were able to determine the mutant and ancestral nucleotides at each site by comparison of the human and chimpanzee orthologues with the *NANOG *gene and the other pseudogenes.

Some of the source-gene mutations should be distinguishable from post-insertion pseudogene mutations in our data as a nucleotide that is identical in a set of older pseudogenes, which then changes to a different nucleotide in a set of younger pseudogenes. Moreover, if possible source-gene mutations can be identified, they can be used to reconstruct the evolutionary history of the pseudogene family, and to some extent the evolutionary history of the gene itself.

To reconstruct the evolutionary history of the *NANOG *gene and its pseudogene family with source-gene mutation analysis, we aligned the reading frame of the human and chimpanzee *NANOG *gene with the corresponding sequences in all pseudogenes (except *NANOGP11*, which lacks the reading frame), and corrected (in all but four cases) post-H/C divergence mutations to reflect the ancestral sequence. We identified sites with possible source-gene mutations as a nucleotide shared by two or more pseudogenes and a different nucleotide shared by two or more additional pseudogenes. Any nucleotide present in a particular position in only one pseudogene was considered as a post-insertion pseudogene mutation. A total of 68 sites (out of 918) within the reading frame met these criteria for identification of possible source-gene mutations. We then identified the most parsimonious order of pseudogenes as the one which required the fewest number of source-gene mutations across these 68 sites.

The most parsimonious ordering of the *NANOG *pseudogenes (154 possible source-gene mutations across 68 sites) from most ancient to most recent is *NANOGP6*, *NANOGP5*, *NANOGP3*, *NANOGP10*, *NANOGP2*, *NANOGP9*, *NANOGP7*, *NANOGP1*, *NANOGP4*, and *NANOGP8 *as the most recent. The next most parsimonious ordering (156 mutations) is the same as the above order but with the positions of *NANOGP5 *and *NANOGP3 *reversed. As a truncated pseudogene, *NANOGP3 *contains only 19 possible source-gene mutation sites. Of these, only five are informative in distinguishing *NANOGP3 *and *NANOGP5*, three supporting *NANOGP5 *as the older pseudogene and two supporting *NANOGP3*. Sites with only one mutation in a particular order are more likely to represent a true source-gene mutation than sites with multiple mutations, which probably consist of a combination of source-gene and post-insertion mutations. The three sites, 399, 531, and 568, that support *NANOGP5 *as the older pseudogene require 1, 2, and 1 mutations to explain the order, respectively. The two sites that support *NANOGP3 *as the older pseudogene (sites 390 and 566) require 5 and 4 mutations, respectively, to explain that order, suggesting that the most parsimonious order (*NANOGP5 *older than *NANOGP3*) is also the most plausible with respect to these two pseudogenes. Additionally, our analysis clarifies the relative order of *NANOGP2 *and *NANOGP9 *by clearly placing *NANOGP2 *as the older of the two (reversing their positions in the order requires 168 mutations).

The only notable discrepancy between the results of source-gene mutation analysis and ordering by overall similarity to the modern *NANOG *gene is the relative placement of *NANOGP1 *and *NANOGP4*. In the latter analysis, the functional *NANOG *gene is more similar to *NANOGP1 *(98.6%) than it is to *NANOGP4 *(96.4%), implying that *NANOGP4 *is the older pseudogene. However, source-gene mutation analysis places *NANOGP4 *as the more recent of the two. Examination of the mutations that distinguish *NANOGP1 *from *NANOG *provides compelling evidence that *NANOGP1 *is indeed the older pseudogene. *NANOGP1 *is an unprocessed pseudogene that arose from duplication of a segment of chromosome 12, and thus may have remained functional for an undetermined period of time after its formation. As Booth and Holland [4] pointed out, *NANOGP1 *cannot use the same initiation codon as *NANOG *because a mutation at position 25 in the reading frame produced a premature termination codon after only eight amino acids. This mutation is present in both the human and chimpanzee orthologues indicating that it preceded the H/C divergence. Booth and Holland noted, however, that of the three characterized human transcripts from *NANOGP1*, two are alternatively spliced to remove all of exon 1, so that the *NANOGP1 *reading frame begins at a position corresponding to the 58^th ^amino acid in the protein encoded by *NANOG*, which is an internal methionine in the NANOG protein. If *NANOGP1 *did indeed remain functional after its formation, we would expect natural selection to conserve the sequence within its reading frame when compared to *NANOG*.

After correction to the ancestral sequence for post-H/C divergence mutations, 15 mutations distinguish *NANOGP1 *from the *NANOG *reading frame, and they are nonrandomly distributed. Twelve are clustered in a 121-nucleotide region entirely within exon 1 of the *NANOG *gene, a region removed during splicing in two characterized *NANOGP1 *transcripts. Of the three mutations in *NANOGP1*'s apparent reading frame, two are nonsynonymous and one is synonymous. A nonsynonymous mutation at position 246 is a guanine-to-thymine substitution that results in a lysine-to-asparagine substitution in the protein. Comparison with the human and chimpanzee sequences of the other pseudogenes reveals that this is a source-gene mutation that supports *NANOGP1 *as being older than *NANOGP4*. Comparison of this polymorphism to the sequences of the other pseudogenes reveals that the guanine in *NANOGP1*, and therefore the lysine in the protein, are ancestral, and that the source-gene mutation occurred after duplication of *NANOGP1 *but before insertion of *NANOGP4*. Interestingly, Booth and Holland [4] found through experimental sequencing that this particular mutation (and amino acid substitution) is polymorphic in modern humans, suggesting that neither lysine nor asparagine is detrimental to protein function at this position.

The other nonsynonymous mutation is a cytosine-to-thymine substitution at position 477, resulting in a proline-to-leucine substitution in the protein. Because proline and leucine have similar biochemical properties, this mutation is also not likely to adversely affect protein function. The *NANOG *gene and all other pseudogenes in both the human and chimpanzee genomes have a cytosine residue at this position, indicating that this is a post-duplication mutation in *NANOGP1*.

The single synonymous mutation in the apparent reading frame is at position 384, which lies within the homeobox region. This is clearly a source-gene mutation that also supports the ordering of *NANOGP1 *as being older than *NANOGP4*. Only *NANOG*, *NANOGP4*, and *NANOGP8 *have a cytosine at this position; all other pseudogenes, including *NANOGP1*, have a thymine at this position.

Taken in the aggregate, these observations strongly support the hypothesis that *NANOGP1 *remained functional after duplication and, therefore, was subject to selection-driven conservation of its reading frame. They also raise the possibility that *NANOGP1 *may retain some functionality or that its loss of function may be evolutionarily recent.

**Figure 2:**
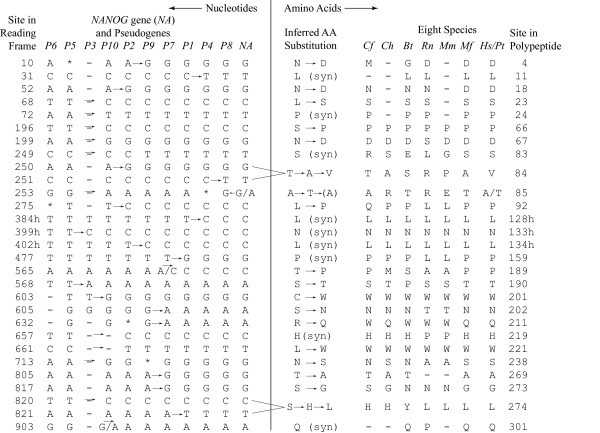
**Potential single source-gene mutations in the most parsimonious ordering of the *NANOG *pseudogenes by source-gene mutation analysis**. The left side depicts nucleotide sequences of the *NANOG *gene and pseudogenes after correction of post-H/C divergence mutations to the ancestral sequence. In two instances (sites 565 and 903), the ancestral sequence could not be determined, so both human and chimpanzee sequences are indicated with the human sequence on the left. At site 253, the human and chimpanzee sequences differ for *NANOG*, and the chimpanzee sequence is ancestral. However, we included the polymorphism because it explains the guanine in *NANOGP8 *(reverse arrow). Asterisks (*) denote post-insertion mutations and hyphens (-) denote deletions in the DNA sequences of the pseudogenes. The right side depicts inferred amino acid substitutions and the corresponding amino acids in the NANOG proteins of eight species: *Cf *= *Canis familiaris*, *Ch *= *Capra hircus*, *Bt *= *Bos taurus*, *Rn *= *Rattus norvegicus*, *Mm *= *Mus musculus*, *Mf *= *Macaca fascicularis*, *Hs *= *Homo sapiens*, *Pt *= *Pan troglodytes*. The "h" designation following a site number indicates that the site lies within the homeobox region.

Nucleotide polymorphisms at possible source-gene mutation sites may represent true source-gene mutations or post-insertion pseudogene mutations. Sites in which a single mutation separates a set of older pseudogenes from a set of younger pseudogenes are the most plausible sites for identification of true source-gene mutations. In the most parsimonious ordering, 29 of the 68 sites contained a single possible source-gene mutation (Figure 2). Twenty of these mutations are nonsynonymous and nine are synonymous. If a mutation is indeed a true source-gene mutation, the amino acid it encodes may be reflected in the *NANOG *proteins of other vertebrates. To determine if this is the case, we used the amino acid sequence of the polypeptide encoded by the human *NANOG *gene [GenBank:NP_079141] as a query for a BLASTP search of the protein database of all organisms. Proteins from six species displayed full-length or nearly full length homology to the *NANOG *protein: crab-eating macaque (*Macaca fascicularis *[GenBank:BAD72891]), house mouse (*Mus musculus *[GenBank:XP_132755]), Norway rat (*Rattus norvegicus *[GenBank:XP_575662]), domestic cattle (*Bos taurus *[GenBank:AAY84556]), domestic goat (*Capra hircus *[GenBank:AAW50709]), and domestic dog (*Canis familiaris *[GenBank:XP_543828]). We excluded a match to a computationally generated hypothetical protein in chimpanzee [GenBank:XP_510125] because it is derived from the DNA sequence of chimpanzee *NANOGP7*.

As shown in Figure 2, several of the putative source-gene mutations and their inferred effect on amino acid sequence in the human/chimpanzee *NANOG *pseudogene family are consistent with the corresponding amino acids in the *NANOG *proteins of other eutherian mammals. For example, at site 52 in the reading frame, an adenine-to-guanine substitution in the *NANOG *gene apparently occurred after the insertion of *NANOGP10 *but before the insertion of *NANOGP2*, resulting in an asparagine-to-aspartic acid substitution in amino-acid residue 18 of the polypeptide. The dog, cattle, and rat proteins have asparagine at this position, whereas the macaque, chimpanzee, and human have aspartic acid at this position. Similar patterns of congruence between amino acid substitution and amino acid sequences in other mammals is evident at positions 250–251, 275, 568, 713, 817, and 820–821 of the reading frame (Figure 2).

Another feature of the putative source-gene mutations is the paucity of amino acid substitutions at source-gene mutation sites within the homeobox region (positions 283–462 in the reading frame) indicative of high source-gene sequence conservation in this region. Six possible source-gene mutation sites are present within the homeobox region (three of which are single-mutation sites depicted in Figure 2). Five of these six sites have only synonymous mutations. The single nonsynonymous mutation is at position 358, with thymine present in *NANOGP7 *and *NANOGP9 *and cytosine present in all other pseudogenes and the *NANOG *gene, resulting in a leucine-to-phenylalanine substitution in the *NANOGP7 *and *NANOGP9 *sequences. These thymines may be independent post-insertion mutations or they could be a source-gene mutation that reverted to its original sequence after the insertion of *NANOGP7*.

Pseudogene mutations can be used to estimate the dates of origin for individual pseudogenes. However, only post-insertion mutations not subject to purifying selection are reliable indicators of the age of a pseudogene. Our analysis shows that, in the case of the *NANOG *pseudogene family, source-gene mutations are present and may contribute to a significant number of polymorphisms in the pseudogenes. Although some source-gene and post-insertion mutations may be readily distinguished based on their patterns when the pseudogenes are ordered, others may not be so easily discerned. Even when post-insertion mutations can be reliably identified, pseudogene evolution rates have not been well calibrated prior to the H/C divergence, as pointed out by Booth and Holland [4]. For these reasons, we have avoided age estimations in this study, focusing instead on the relative order of *NANOG *pseudogene origins.

## Conclusion

A synthesis of the results from this article with those of Booth and Holland [4] produces a straightforward evolutionary history of the *NANOG *pseudogene family in the human and chimpanzee genomes. *NANOGP6 *is the most ancient of the pseudogenes followed in order of most ancient to most recent by the processed pseudogenes *NANOGP5*, *NANOGP3*, *NANOGP10*, *NANOGP2*, *NANOGP9*, *NANOGP7*, and *NANOGP4*. Before insertion of *NANOGP4*, the region on chromosome 12 containing *NANOG *underwent a duplication producing *NANOGP1*, which remained functional and subject to selection-driven conservation of its reading frame. All of these events, and the resulting fixation of their products in the genome, preceded the H/C divergence. Following the H/C divergence, *NANOGP8 *inserted itself into chromosome 15 in the human lineage.

## Methods

### DNA amplification, cloning, and sequencing

We obtained chimpanzee DNA (individual PR00226) from the Integrated Primate Biomaterials and Information Resource (IPBIR) of the Coriell Institute for Medical Research (Camden, NJ, USA). We selected sequences for PCR primers specific to the chimpanzee *NANOG *gene by comparing the *NANOG *and *NANOGP1 *sequences from the Build 1.1 assembly and selecting sites with at least two variant nucleotides, with a variant nucleotide on the 3' end of each primer. We selected primer sequences for the *NANOGP9 *reading frame by identifying sites that contained two variants unique to human *NANOGP9 *with a variant nucleotide on the 3' end of each primer. All oligonucleotide primers were manufactured by Integrated DNA Technologies, (Coralville, IA, USA). We amplified DNA using AccuprimeT Hi-Fidelity *Taq *polymerase (Invitrogen, Carlsbad, CA, USA) according to the manufacturer's recommendation at 2.5 mM MgCl_2_. The PCR amplification protocol consisted of an initial denaturation step of 1.5 min at 94°C, followed by 35 cycles of amplification consisting of 30 s denaturation at 94°C, 30 s for primer annealing at 57°C and between 1 and 5 min of extension at 68°C, depending on the anticipated product size (1 min/1 kb). We cloned the resulting amplicon using the pGEM-T Easy Vector System II (Promega, Madison, WI, USA), and identified recombinant clones by standard blue/white screening methods with IPTG and X-Gal. We purified plasmid DNA from each selected recombinant clone using a GenEluteTM plasmid miniprep Kit (Sigma, St. Louis, MO, USA) and quantified the DNA using a spectrophotometer. Isolated plasmid DNA was sequenced bidirectionally from M13 (F/R) primers. A 3,889 nucleotide-pair clone containing exon 1, intron 1 and part of exon 2 of the *NANOG *gene was sequenced by primer walking. DNA sequencing was performed at the Brigham Young University DNA Sequencing Center (Provo, UT, USA) using standard ABI Prism Taq dye-terminator cycle-sequencing methodology. DNA sequence chromatograms were analyzed with the Contig Express program in the Vector NTI software suite (InforMax, Frederick, MD, USA).

### DNA sequence analysis

To initially identify the locations and DNA sequences of the *NANOG *gene and its pseudogenes in the chimpanzee genome, we used the GenBank entries for the human *NANOG *gene and its 11 pseudogenes as queries for MEGABLAST searches of the chimpanzee genome Build 1.1 assembly with default settings including filtering for repetitive sequences. After identifying the genes and pseudogenes in the chimpanzee genome, we copied the sequences and alignments then confirmed and refined them with the "align two sequences" (bl2seq) BLAST tool with a word size of seven and filtering disabled. We further refined the alignments manually, especially on the ends of the sequences where word-size limitations failed at times to identify true alignments.

We copied flanking DNA sequences on both sides of the human *NANOGP8 *and *NANOGP9 *pseudogenes and used them as MEGABLAST queries with default settings and filtering to search the chimpanzee genome to confirm whether or not these pseudogenes were present. After MEGABLAST identified the sequences, we refined alignments with the bl2seq tool with a word size of seven and filtering disabled and with manual refinements.

To facilitate determination of the evolutionary order of pseudogene origin, we copied the reading frame of the functional human *NANOG *gene and used it as a query in the bl2seq tool with a word size of seven and filtering disabled to determine the best alignment with the corresponding regions of each of the pseudogenes except *NANOGP11*, which does not include the reading-frame region. We used these alignments to generate a multiple alignment of the reading-frame region of human and chimpanzee orthologues of the *NANOG *gene and all pseudogenes except *NANOGP11*. This multiple alignment allowed us to identify post-H/C divergence mutations and correct them to reflect the ancestral sequences, and to identify and distinguish between potential source-gene mutations and post-insertion mutations in the pseudogenes, as described in the results and discussion section.

## List of abbreviations

UTR = untranslated region

H/C divergence = human-chimpanzee divergence

EST = expressed sequence tag

## Authors' contributions

DJF carried out all BLAST searches, sequence alignments, and evolutionary analyses. PJM carried out all DNA amplification, cloning, and experimental sequence assemblies. Both authors drafted the manuscript.
